# Unraveling Resistance in Lung Cancer Immunotherapy: Clinical Milestones, Mechanistic Insights, and Future Strategies

**DOI:** 10.3390/ijms26189244

**Published:** 2025-09-22

**Authors:** Maria Vitale, Raffaella Pagliaro, Giuseppe Viscardi, Lucio Pastore, Giuseppe Castaldo, Fabio Perrotta, Susan F. Campbell, Andrea Bianco, Filippo Scialò

**Affiliations:** 1 CEINGE-Biotecnologie Avanzate Franco Salvatore, 80145 Naples, Italy; vitalema@ceinge.unina.it; 2 Department of Translational Medical Sciences, University of Campania L. Vanvitelli, 80131 Naples, Italy; raffaella.pagliaro@studenti.unicampania.it (R.P.); fabio.perrotta@unicampania.it (F.P.); sfmcampbell@gmail.com (S.F.C.); 3 Department of Pneumology and Oncology, Monaldi Hospital, AORN dei Colli, 80131 Naples, Italy; giuseppe.viscardi@hotmail.it; 4 Department of Molecular Medicine and Medical Biotechnologies, University of Naples Federico II, 80131 Naples, Italy; lucio.pastore@unina.it (L.P.); giuseppe.castaldo@unina.it (G.C.); 5 U.O.C. Clinica Pneumologica L. Vanvitelli, Monaldi Hospital, AORN dei Colli, 80131 Naples, Italy

**Keywords:** lung cancer, immunotherapy, immune checkpoint inhibitors, resistance mechanisms, biomarker, personalized medicine

## Abstract

Over the last decade, immunotherapy has revolutionized lung cancer treatments, particularly in non-small cell lung cancer, where immune checkpoint inhibitors have achieved significant clinical success. However, high percentages of patients do not respond initially or eventually develop a resistance to these therapies. This review explores the evolution and challenges of immunotherapy in lung cancer, highlighting its clinical milestones and intrinsic and extrinsic resistance mechanisms. We investigate tumor-intrinsic resistance factors, including alterations in antigen presentation, the loss of Beta-2 microglobulin function, impaired interferon signaling, immune editing, epigenetic modifications, and tumor-extrinsic resistance, such as an immunosuppressive lung tumor microenvironment, dysregulated cytokine profiles, and the upregulation of immune checkpoints. Then, we focus on the emerging role of resistance biomarkers and the development of personalized treatment strategies to overcome these challenges. The complex interplay between tumor biology and immune modulation in lung cancer paves the way for novel approaches for improving the effectiveness of immunotherapeutic treatments.

## 1. Introduction

The immune system plays a central role in controlling tumor development through a dynamic process often described as the cancer–immunity cycle. In this cycle, tumor-derived antigens are released by dying cancer cells and captured by antigen-presenting cells (APCs), particularly dendritic cells. These APCs migrate to lymph nodes, where they activate cytotoxic CD8^+^ T lymphocytes that recognize tumor-associated antigens. Activated T cells then infiltrate the tumor microenvironment, recognize cancer cells through major histocompatibility complex (MHC)–peptide interactions, and induce cell death. Natural killer (NK) cells also contribute to this surveillance by targeting cells with reduced or absent MHC expression [1].

The introduction of immunotherapy in cancer treatment has marked a revolutionary turning point in the fight against cancer, enabling the immune system to be exploited as a powerful and effective weapon against tumor cells [1]. Immunotherapy is rooted in the observation that immune cells play a pivotal role in tumor suppression and progression [2,3]. Immunotherapeutic approaches have evolved into one of the most exciting frontiers in modern medicine. Some observations indicate that a rapid and focused immune response may identify and eliminate tumor cells by treating them as foreign invaders [4,5]. Therefore, the primary objective of immunotherapy is to potentiate and amplify the body’s natural defenses, countering the ingenious strategies cancer cells use to evade detection [6]. Despite challenges such as variable response rates and an incomplete understanding of some mechanisms, the field has achieved remarkable milestones [6]. Immune checkpoint inhibitors (ICIs) have revolutionized the treatment landscape for cancers, in particular for melanoma and non-small cell lung cancer (NSCLC), by releasing the brakes on T cell activity [7,8,9,10,11]. The clinical advances regarding the ICIs are grounded in a deeper understanding of the interactions between the immune system and tumor cells. In physiological conditions, the immune system, via anti-tumor immunity, can recognize tumor-associated antigens presented by APCs, prime cytotoxic T lymphocytes, and eliminate tumor cells. However, lung cancer cells frequently evade immune surveillance through multiple mechanisms, including the overexpression of PD-L1, the recruitment of regulatory T cells, and the secretion of immunosuppressive cytokines. These complex processes induce a shutdown of T cell activity, facilitating tumor progression. Immunotherapy, especially ICIs, can restore T cell cytotoxic activity, reactivating the cancer immunity cycle. This biological rationale underpins the clinical benefits observed with Programmed Cell Death Protein 1 (PD-1) and their ligand PD-L1 or Cytotoxic T-lymphocyte antigen 4 (CTLA-4). For example, inhibitors targeting Cytotoxic T-lymphocyte antigen 4 (CTLA-4), such as ipilimumab, enhance T cell priming and activation, significantly improving the overall survival in patients with advanced melanoma [12]. Similarly, Programmed Cell Death Protein 1 (PD-1) inhibitors and their ligand PD-L1 have demonstrated efficacy in various cancers, including NSCLC, renal cell carcinoma, and head and neck cancer, by reactivating exhausted T cells [13,14]. In NSCLC first-line treatments include the use of pembrolizumab (anti PD-1, Keytruda) alone or in combination with chemotherapy, which is approved for both squamous and non-squamous subtypes [11,15]. Several anti-PD-1/PD-L1 agents have been approved by the FDA for the treatment of advanced non-small cell lung cancer (NSCLC), both as first-line therapies (alone or in combination with chemotherapy or other ICIs) and in later lines of treatment. Among these, combinations involving anti-CTLA-4 antibodies, such as ipilimumab (e.g., nivolumab plus ipilimumab in the CheckMate 9LA trial) and durvalumab (e.g., durvalumab plus tremelimumab in the POSEIDON trial), have shown efficacy as first-line options. The detailed information on approved indications and corresponding clinical trials is summarized in Table 1 and Table 2. This review examines the evolution of immunotherapy in LC treatment, focusing on key clinical advancements, mechanisms of resistance, and emerging biomarkers that may predict responses to ICIs.

## 2. Tumor Escape and Mechanism of Resistance: Primary and Acquired Resistance

Although immunotherapy has revolutionized cancer treatments, its transformative potential is tempered by significant limitations, especially in LC patients [16,17]. Only a subset of patients experience long-term benefits from immunotherapy (20–30% of patients treated), mainly due to the heterogeneity of lung tumors and the ability to develop mechanisms of immune evasion [7,16]. The mechanism of tumor escape, whereby cancer cells adopt strategies to avoid immune detection, represents a key challenge. Tumor cells downregulate or alter the expression of antigens, impairing the recognition by cytotoxic T cells. Furthermore, tumor cells upregulate inhibitory molecules such as PD-L1, dampening the immune response and modifying the tumor microenvironment to create a hostile and immunosuppressive niche that may contribute to anti-tumor immunity and tissue homeostasis [18,19]. Primary and acquired resistance are the two main mechanisms of immunotherapy resistance related to lung tumors [20,21]. Currently, a universally accepted definition of resistance to ICIs for clinical practice is lacking. However, the Society for Immunotherapy of Cancer (SITC) consensus proposed a structured framework distinguishing between primary resistance (progressive disease (PD) as best response despite adequate drug exposure or stable disease (SD) for less than six months) and secondary resistance (PD after initial response or SD lasting more than six months). According to Schoenfeld et al., primary resistance encompasses the evidence of PD as the best response, whereas acquired resistance requires an initial response to treatment [22]. Primary resistance is often linked to intrinsic factors, such as a low tumor mutational burden (TMB) and insufficient neoantigen expression [23]. Without these neoantigens, the immune cells cannot recognize the tumor as foreign, rendering ICIs less effective [7]. Another cause of primary resistance could be extrinsic factors such as the poor immune infiltration in the tumor microenvironment (TME), resulting in a ‘cold’ or ‘desert’ microenvironment [24,25]. In such cases, the TME does not have enough activated T cells to sustain an effective immune response, even with ICIs [26]. As a consequence, the early crossing of survival curves observed in many randomized clinical trials (RCTs) suggests the existence of an excess of mortality in the ICI arm compared to the standard of care [27].

Unlike primary resistance, hyperprogressive disease (HPD) is characterized by an accelerated tumor growth rate (TGR) following the initiation of immunotherapy, often accompanied by rapid clinical deterioration. Various definitions have been proposed, but most rely on radiological criteria such as a ≥2-fold increase in the TGR compared to baseline. HPD is particularly challenging as it mimics aggressive disease progression yet appears to be treatment-related. Its incidence varies across studies of immunotherapy in solid tumors, ranging from 4% to 29%, depending on the tumor type, patient population, and diagnostic criteria used [28]. Mechanistically, HPD is still poorly understood, though hypotheses include the expansion of immunosuppressive cell populations, oncogenic pathway activation, or paradoxical immune modulation [28].

In other cases, patients for the first 6 months are responsive to the immunotherapy, with tumor shrinkage or stabilization, but relapse later [29]. When acquired resistance occurs after the exposure to immunotherapy, some cancer cells may lose or alter the specific antigens targeted by T cells [25]. The ‘antigen loss’ means that even though the immune system was initially effective, the tumor cells express a different antigenic profile stimulated by selective pressure, and the immune cells cannot recognize it. The main mechanism of acquired resistance includes a mutation in antigen-presenting machinery, such as mutations in B2-microglobulin or MHC molecules, which prevent T cells’ recognition of tumors, allowing immune evasion [30]. Other acquired resistance mechanisms are related to changes in the TME; over time, LC cells alter their microenvironment to become more immunosuppressive, secreting cytokines like TGF-B, IL-10, and so on. These cytokines recruit regulatory T cells or modify metabolic pathways to inhibit immune cell functions. The resulting environment actively suppresses the anti-tumor immune response [31,32]. In some cases, even if inhibitory receptors such as PD-1 are blocked, tumor cells upregulate other inhibitory receptors as a compensatory strategy, leading to T cell suppression despite using a single immunotherapeutic agent [13,33]. In a study of NSCLC patients with acquired resistance to anti-PD-(L)1 therapy, Ricciuti et al. analyzed matched pre- and post-ICI tumor biopsies and identified recurrent genomic alterations—including mutations in *STK11*, *B2M*, *JAK1/2*, *KEAP1*, and *MTOR*—emerging only after immunotherapy exposure. These changes were associated with an immune desert phenotype, characterized by reduced CD8^+^ T cell infiltration, decreased PD-1/PD-L1 engagement, and the downregulation of HLA class I expression. Specifically, KEAP1 is a negative regulator of NRF2, a transcription involved in the antioxidant response. In physiological conditions, the interaction between KEAP1 and NRF2 promotes its ubiquitination and degradation. Commonly in lung cancer lung adenocarcinoma, KEAP1 is mutated or inactivated, stabilizing NRF2 that translocates into the nucleus, sustaining the antioxidant and cytoprotective gene program. Additionally, the stabilization of NRF2 promotes the secretion of immunosuppressive cytokines and supports the recruitment of MDSCs and TAMs, dampening anti-tumor immunity.

**Figure 1:**
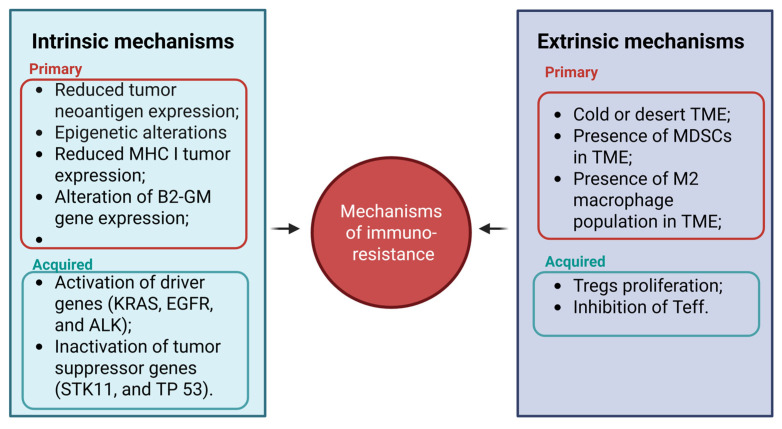
Intrinsic and extrinsic mechanisms of immune resistance. Multiple mechanisms contribute to both intrinsic and extrinsic immune resistance, significantly impacting the efficacy of immunotherapy. Intrinsic mechanisms involve tumor cell-autonomous factors, while components of the tumor microenvironment mediate extrinsic mechanisms. Understanding these pathways is critical for improving immunotherapeutic outcomes. In each box, the mechanisms related to primary and acquired immunoresistance are indicated in red and green, respectively.

The primary and acquired resistance explain why some subsets of LC patients treated with immunotherapy showed long-term benefits and underline the ongoing need to evaluate combinatorial therapy, novel agents, predictive biomarkers, and personalized medicine to overcome the resistance mechanisms [34] (Figure 1).

## 3. Tumor-Intrinsic Resistance in Lung Cancer

As previously mentioned, several key factors mainly contribute to the tumor’s intrinsic resistance, limiting the effectiveness of current immunotherapeutic approaches. The mechanism of tumor-intrinsic resistance includes an extensive range, from antigen presentation alterations to poor antigen expression, the activation of driver genes, and the tumor-mediated silencing of the immune system via cytokine release [7]. Tumor cells often present antigens that are not found in normal cells. These tumor-specific antigens (TSAs) can originate from genetic mutations, viral infections, or other changes unique to the tumor environment. A subset of TSAs, neoantigens, specifically arises from somatic mutations within tumor cells. These mutations lead to the formation of new peptide sequences that the immune system can recognize as foreign, distinguishing the tumor from healthy tissue. Furthermore, TSAs efficiently activate T cells to recognize tumors as non-self, initiating the immune response and mediating immune tumor killing [35]. A certain type of tumor could express a low number of TSAs, resulting in a reduction in T cell activation and subsequently a reduction in tumor immunogenicity [23,36,37]. This aspect is considered a resistance marker to immunotherapy. It has been noted that NSCLC and melanoma have higher mutation burdens and are more sensitive to immunotherapy compared to pancreatic cancer and thyroid carcinoma, which have lower mutation burdens and exhibit a lower sensitivity to immunotherapy [7]. Another character involved in the tumor-intrinsic resistance mechanism is the major histocompatibility complex I (MHC I) [30]. Tumor cells can disrupt the antigen presentation by reducing the expression of MHC I receptors, which are crucial for displaying tumor-derived antigens [12]. They may also impair the function of antigen-processing components, such as TAP1/2 and β2-microglobulin (B2-GM), further hindering the immune system’s ability to recognize and target the tumor. B2-GM is one of the main proteins that compose the heavy chain of MHC I, responsible for folding and transporting MHC I on the plasma membrane [6,38,39]. The alteration in the B2-GM gene (B2M) leads to a reduction in MHC I on the cell surface that, in turn, prevents effective recognition by cytotoxic T lymphocytes, contributing to immune evasion in NSCLC [40,41].

### Driver Genes and Inactivation of Tumor Suppressor Genes Contribute to Intrinsic Resistance in Lung Cancer

**Figure 2:**
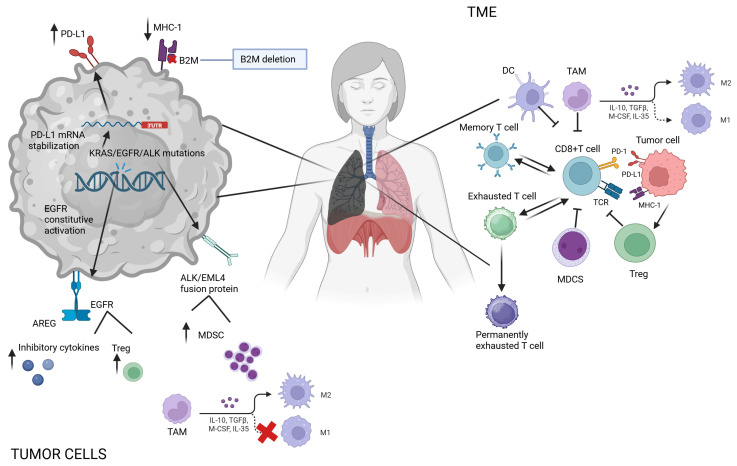
Mechanisms of resistance to immune therapy. The mechanism of tumor-intrinsic resistance includes antigen presentation alteration, poor antigen expression, activation of driver genes, and tumor-mediated silencing of the immune system via cytokine release. Tumor cells reduce MHC I surface expression by downregulating components like TAP1/2 and β2-microglobulin (B2M), impairing antigen presentation and cytotoxic T cell recognition. Mutations in driver genes such as KRAS, EGFR, and ALK further enhance immune escape. KRAS mutations upregulate PD-L1, while EGFR activation promotes Treg cell expansion and inhibitory cytokine release. ALK-EML4 fusion protein induces an increase in myeloid-derived suppressor cells (MDSCs) and M2 macrophages transition. Tumor-extrinsic resistance is primarily driven by the tumor microenvironment (TME). Regulatory T cells (Tregs) suppress immune responses by releasing inhibitory cytokines (IL-10, TGF-β, IL-35) that downregulate MHC-II and co-stimulatory molecules on dendritic cells (DCs), impairing effector T cell (Teff) activation. Myeloid-derived suppressor cells (MDSCs) promote immune evasion and tumor progression, and their accumulation in the TME is correlated with reduced immunotherapy efficacy. Tumor-associated macrophages (TAMs) are classified into M1 (anti-tumor) and M2 (pro-tumor) types. M2 macrophages, attracted by chemokines (CCL2–5) and cytokines, suppress T cell activity and secrete IL-10 and TGF-β, further contributing to immune suppression.

In LC, several driver genes involved in tumor progression also contribute to immune escape, including Kirsten rat sarcoma virus oncogene homolog (KRAS), epidermal growth factor receptors (EGFRs), and anaplastic lymphoma kinase (ALK) [42,43]. For instance, KRAS G12C mutations have been linked to elevated PD-L1 expression, as they stabilize the 3’ UTR region of PD-L1 mRNA, thereby enhancing the tumor’s ability to evade the immune system. The EGFR is the most commonly mutated gene in NSCLC, and several clinical trials showed that the patients who carry this mutation do not respond well to immunotherapy [44,45]. The mutation that affected the EGFR induces its constitutive activation, and the ligand named amphiregulin (AREG) stimulates the production of regulatory T cells (Tregs) via the EGFR–glycogen synthase kinase-3B-forkhead box P3 axis, enhancing immunosuppression [46]. Additionally, the EGFR signal can stimulate the production of inhibitory cytokines that stimulate the proliferation of myeloid-derived suppressor cells and tumor-associated macrophages and inhibit cytotoxic T cell responses [47]. Furthermore, the signal transducer and activator of transcription 3 (STAT3), a downstream factor of the EGFR signal pathway, is up regulated and guides the decrease in MHC I expression, which is already described and contributes to immune evasion. The effects of the STAT 3 activation are numerous and also mediate the expression of vascular endothelial growth factors (VEGFs), interleukin-6 (IL-6), and interleukin-10 (IL-10) and prevent the differentiation and maturation of dendritic cells (DCs) [48,49]. Chromosomal reorganization involving ALK leads to the fusion of the ALK and EML4 genes [50]. The resulting tyrosine kinase not only reduces the production of new tumor antigens but also promotes the generation of immunosuppressive cells through the activation of the PI3K-AKT and MEK-ERK signaling pathways [51]. This dual effect ultimately diminishes the efficacy of immunotherapy. Accordingly, outcomes for patients with actionable driver mutations (*EGFR*, *ALK*, *ROS1*, *RET*) are unsatisfactory, and ICIs should only be considered after the exhaustion of targeted therapies and salvage chemotherapies [52]. The immune checkpoint inhibition efficacy can also be affected by the inactivation of tumor suppressor genes. Mutations in the STK11 gene are observed in approximately 8% to 39% of patients with NSCLC; this gene negatively regulates the mTOR signaling pathway, which is involved in several essential cellular processes such as nutritional signals, the regulation of protein synthesis, cell growth, and metabolic modulation [53,54]. Mutations in STK11 lead to a decreased infiltration of cytotoxic CD8+ T lymphocytes sustaining a ‘desert’ TME [55]. *STK11* and *KEAP1* mutations confer worse outcomes with immunotherapy among patients with *KRAS*^MUT^ but not among patients with *KRAS*^WT^ [56], provoking an accrual of neutrophils with T cell suppressive effects and tumor-promoting cytokines [16,55]. Beyond the direct effects of SKT11, KRAS, and TP53 mutations on the resistance to immunotherapy, epigenetic alterations, including DNA methylation, histone modification, and non-coding RNA expression, can further modulate gene expression and contribute to the resistance to immunotherapy [57]. The expression of ICIs may be affected by epigenetic modifications, disrupting the antigen presentation process and inhibiting the migration and activation of T cells at the tumor site [58] (Figure 2).

## 4. Tumor-Extrinsic Resistance

**Figure 3:**
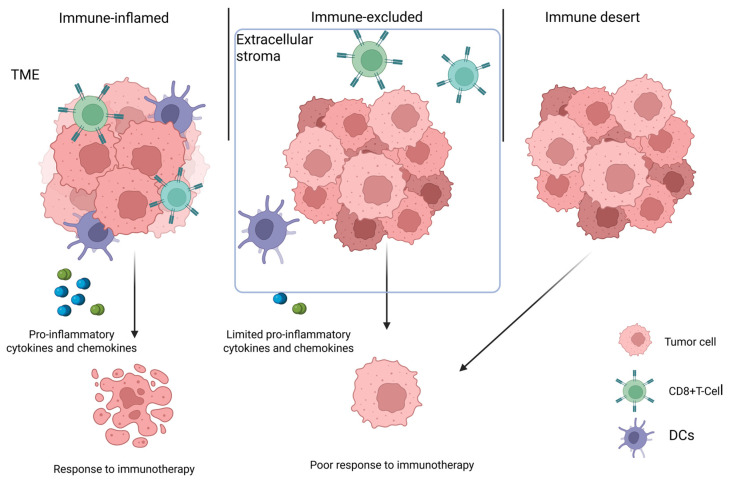
Tumor immunophenotype involved in extrinsic immune resistance. TME immune phenotype has a pivotal role in immunotherapy tumor response. The tumor immune phenotype could be immune-inflamed, immune-excluded, or immune-deserted. The immune-inflamed TME is characterized by significant infiltration of CD8+ T cells and exhibits high levels of pro-inflammatory cytokines and chemokines. The immune-excluded TME is characterized by the presence of numerous immune cells confined to the surrounding stroma, and the desert TME is an environment that lacks significant immune cell infiltration and produces minimal inflammatory signals.

Tumor-extrinsic resistance is primarily driven by the TME, an intricate network of cells and signals that support tumor growth. The modulation of the TME is directly linked to tumor progression and development [21,59]. Among tumor-infiltrating lymphocytes (TILs), CD8+ T cells have a strong anticancer activity by eliminating tumor cells directly, and their distribution is essential to define the immune phenotype of the cancer [60,61,62]. Specifically, the tumor immune phenotype could be immune-inflamed, immune-excluded, and immune-deserted. The immune-inflamed TME is characterized by the significant infiltration of CD8+ T cells and exhibits high levels of pro-inflammatory cytokines, chemokines, and other immune mediators. The infiltration of active immune cells is linked to a favorable response to immunotherapy. However, another resistance phenotype, known as immune exclusion, is characterized by the presence of numerous immune cells within the TME. Despite their abundance, these cells remain confined to the surrounding stroma and are unable to penetrate the tumor itself [63]. As a result, the anti-tumoral activity of immune cells is limited, and this type of tumor does not completely respond to immunotherapy. As the word ‘desert’ suggests, the immune desert environment refers to a tumor environment that lacks significant immune cell infiltration and produces minimal inflammatory signals [64]. As with the immune-excluded phenotype, the immune desert TME is also generally poorly responsive to immunotherapy, and strategies to convert the TME from ‘cold’ to ‘hot’ are a major area of ongoing research (Figure 3).

### Main Cells Involved in Tumor-Extrinsic Resistance

Although the immune phenotype of the TME plays a pivotal role in modulating immune response efficacy, other cells also contribute to immunosuppression. Among them, regulatory T cells (Tregs) [65], myeloid-derived suppressor cells (MDSCs) [66], and M2 macrophages directly influence the immune response modulation [67]. Tregs are a specialized subset of CD4+ T cells that play a pivotal role in maintaining immune tolerance and preventing autoimmune disease. Tregs exert their action by secreting inhibitory cytokines such as IL-10, IL-35, and TGF-B; IL-10 secretion especially influences antigen presentation by downregulating the MHC-II expression and co-stimulatory components on DCs, in turn inhibiting Teff activation. The ratio between Teff and Tregs is closely related to ICI responses, and the factors that stimulate Treg proliferation are presumptive biomarkers of resistance with a poor prognosis [68,69]. The elimination or reduction in Tregs at the tumor site should be an essential part of treatments. Another critical modulator of the immune response in cancer are the MDSCs. MDSCs promote immune evasion and tumor growth, and several studies have demonstrated that the presence of these cells in the TME leads to a reduction in the efficacy of immunotherapies, while reprogramming or reductions improve clinical responses to immunotherapy [70]. The circulating macrophage and monocyte recruitment at the tumor site could be involved in tumor progression. The tumor-associated macrophages (TAMs) are classified into two classes, M1 and M2, according to the expression of surface molecules, the cytokine profile, the expression of transcription factors, and metabolism [71]. M1 has an anti-tumor effect, while M2 has pro-tumorigenic properties, modifying the TME. The TAMs are attracted by the chemokine production at the tumor site, such as CCL2, CCL3, CCL4, CCL5, and cytokines. M2 exerts its action on T cells by de-potentiating T cells’ ability for antigen presentation and secreting IL-10 and TGF-B, which have an immunosuppressive function [72] (Figure 2). Beyond tumor-intrinsic mechanisms, a growing body of evidence emphasizes the impact of host- and environment-related factors on response to ICIs by creating an immunosuppressive microenvironment through Tregs, MDSCs and M2 macrophages, which inhibit T cell activation. In addition, the gut microbiome is a key modulator, influenced not only by the microbial composition but also by diet, which can alter metabolite production and immune priming [73]. Moreover, sarcopenia—often associated with chronic inflammation and impaired immune competence—has been linked to worse outcomes in ICI-treated patients. Concomitant infections, as well as the use of certain concomitant medications, notably antibiotics and proton pump inhibitors (PPIs), have been shown to negatively affect ICI efficacy, likely through disruption of the gut microbial balance or the modulation of systemic immunity [74]. Recognizing and addressing these host-related influences may improve patient selection and therapeutic personalization.

In the context of the ICIs, tumor-extrinsic resistance plays a critical role in limiting the efficacy of immune checkpoint inhibitors. Indeed, even if they can act on PD-1/PD-L1 or CTLA-4, the therapy is ineffective if T cells fail to infiltrate the tumor, which happens in the desert or immune-excluded TME. Unfortunately, an immunosuppressive tumor microenvironment—characterized by regulatory T cells, myeloid-derived suppressor cells, tumor-associated macrophages, suppressive cytokines (e.g., TGF-β, IL-10, VEGF), metabolic competition, and stromal barrier—can prevent effective T cell infiltration and function. These extrinsic factors undermine the reinvigoration of anti-tumor immunity by ICIs, leading to primary or acquired resistance despite checkpoint blockades.

## 5. Resistance Biomarkers and Personalized Treatments

LC is one of the leading causes of cancer-related mortality worldwide [75,76]. Although advances in customized therapy and immunotherapy have been made, treatment resistance remains one of the major challenges. The identification of resistance biomarkers is crucial for predicting therapy responses and the development of personalized treatments. In LC, particularly in NSCLC, the most clinically established biomarkers are PD-L1 expression and TMB [7,77]. As reported previously, PD-L1 and its receptor PD-1 are involved in downregulating the immune response and self-tolerance [78]. Certain types of tumor cells, including those in NSCLC, can upregulate PD-L1 expression, suppressing anticancer immune activity. The interaction with PD-1 can be counteracted through ICIs [79]. To date, seven ICIs have been approved by regulatory agencies for the treatment of LC. The primary method for assessing PD-L1 expression in tumors is immunohistochemistry (IHC), which quantifies expression using the tumor proportion score (TPS) [80]. The TPS is determined by evaluating both the percentage of tumor cells expressing PD-L1 on their surface and the intensity of the staining [80,81]. The prediction of the immunotherapeutic response mediated by PD-L1 expression is well established; high levels of PD-L1 correlate with an improvement in the ICI response rate and superior survival, while low or negative PD-L1 expression is associated with immunotherapy resistance. PD-L1 is an effective predictive biomarker in the treatment of NSCLC and is the only biomarker used routinely in clinical practice to orient immunotherapy treatment decisions [82]. Although IHC is a relatively simple and efficient technique, the use of PD-L1 as a biomarker presents several limitations [81]. Notably, evaluating PD-L1 expression alone is not sufficient to reliably predict the efficacy of immunotherapy. One major limitation is the assay variability, as numerous staining protocols and antibody clones are available, resulting in inconsistencies across different platforms. To address this lack of standardization, the IASLC BluePrint IHC Comparability Project identified three assays, 22C3, 28-8, and SP263, that demonstrated comparable TPSs [81]. The second pivotal limitation is related to the spatial and temporal heterogeneity of PD-L1 expression. The PD-L1 expression level depends on the location of the tissue sample, such as a primary tumor, metastatic site, or others. Furthermore, expression levels may also differ during the disease course and in response to specific treatments [83,84]. However, in the management of NSCLC, the evaluation of PD-L1 expression is the main approach for establishing treatments but remains inaccurate in terms of predictive value. In addition, several studies have reported the association between the TMB and ICIs in NSCLC [84,85]. The TMB indicates the number of somatic non-synonymous mutations in the genome of the tumor, including substitutions and short insertions/deletions [86]. A high TMB is typically associated with stronger and more durable immunotherapy responses, which improve survival outcomes [87]. A high TMB results in a larger number of antigens exposed on the surface of tumor cells, which increase the immune recognition and consequently the tumor becomes more sensitive to treatments with immunotherapy. Thus, a high TMB is a positive biomarker of the immunotherapy response and survival [88]. The evaluation of the TMB is performed by whole-genome sequencing (WGS) and targeted panel sequencing via next-generation sequencing (NGS). Despite WES being costly, time-consuming, and needing large tissue samples for comparison, it is the most accurate technique for TMB evaluations [85,89]. As for PD-L1-predictive biomarkers, the TMB also has some limitations; a high TMB is not always predictive of a superior immunotherapy response, and similarly, a low TMB cannot definitively exclude ICI therapy [90]. Furthermore, the platforms used to evaluate the TMB typically present significant variability, with disparate panel sequence sizes, mutation types and numbers, sample requirements, and output capabilities [85]. Another important limiting factor related to the TMB as a biomarker is the variability of thresholds to determine a ‘high TMB’ status [86]. Across various clinical trials, different thresholds have been used; however, the FDA recommends a cut-off of ≥10 mutations/Mb to support the use of ipilimumab and nivolumab for the treatment of NSCLC [91]. An interesting serum-based biomarker is the neutrophil-to-lymphocyte ratio (NLR), which is calculated by dividing the absolute neutrophil count by the absolute lymphocyte count. An elevated NLR has been associated with a TME characterized by high neutrophil and low lymphocyte infiltration. This immunological profile promotes angiogenesis, inhibits apoptosis, and supports tumorigenesis, ultimately correlating with poorer clinical outcomes. In patients with NSCLC receiving immunotherapy, a high baseline NLR is emerging as a potential negative prognostic marker [92,93,94]. Despite its ease of calculation, the current evidence is primarily derived from small, retrospective studies. Therefore, prospective validation studies are essential to confirm the NLR’s prognostic value in this setting. Emerging technologies, including single-cell phenotyping and high-throughput transcriptomics, have enabled a deeper characterization of the TME and the identification of novel biomarkers, such as ribosomal protein L13a (RPL13A) and guanine nucleotide-binding protein-like 3 (GNL3), which are associated with ICI resistance and may serve as potential targets for combination therapies.

## 6. Discussion

To date, PD-L1 expression assessed by IHC remains the most widely used biomarker for predicting responses to ICIs in NSCLC, but its usefulness is tempered by technical and biological challenges [95]; its limitations include intra- and inter-tumor heterogeneity as well as the subjectivity inherent in pathologist interpretations [16]. The discovery and integration of additional biomarkers, such as the TMB, represent a promising avenue for enhancing the precision of immunotherapy treatments decisions. The TMB may be considered as a surrogate biomarker for the neoantigen load [96] and may enhance immune recognition and T cell responses [97] but is an inconsistent predictor of responses to ICIs in some LC subpopulations. Nevertheless, high TMB levels could be used as a complementary biomarker alongside PD-L1 expression [98]. Moreover, a deficiency in the mismatch repair system (dMMR) leads to microsatellite instability (MSI), which is an emerging indicator for patients potentially responsive to ICI therapy [99]. However, recent evidence suggests that the detection of tumor-derived DNA is essential to activate the immune response triggered by dMMR, thereby revealing a novel approach for the biomarkers.

A therapeutic approach recently explored in the treatment of cancer is chimeric antigen receptor-engineered T cell therapy (CAR-T). This strategy is based on engineered T cells that recognize and target specific antigens on tumor cells, bypassing the need for MHC molecule presentation [100,101]. Although CAR-T cells have shown significant success in treating hematological malignancies, their effectiveness in solid tumors and especially in LC is hindered by TME-related factors [102]. In the TME, CAR-T cells are always exposed to cancer-related signals [103], which over time leads to exhaustion and a decline in their function, a phenomenon known as CAR-T cell exhaustion [104]. Recent studies have identified CD38 as a signature of CAR-T cell exhaustion. This transition of effector T cells into an exhausted state is accompanied by significant epigenetic changes with an increased expression on the surface of these cells of inhibitory receptors such as CTLA-4, PD-1, LAG-3, and TIM-3 [105]. In particular, M2-type TAMs in the TME induced CAR-T to overexpress PD-1, influencing the PD-1/PD-L1 axis and reducing the activity of CAR-T cells. The use of anti-PD-L1 drugs such as atezolizumab has been shown to enhance the anti-tumor activity of CAR-T cells by promoting the apoptosis of type 2 TAMs. Therefore, ICIs can boost CAR-T cell activity by modulating the PD-1/PD-L1 axis and the TME, mitigating CAR-T cell exhaustion [106]. However, combining CAR-T therapy with PD-1 inhibitors should be performed carefully; indeed, using PD-1 inhibitors too early can reduce their ability to block the PD-1 pathway, so it is important to choose the right time for treatment to obtain the best results.

This dynamic and multifaceted approach underscores the versatility of immunotherapy and its potential to redefine cancer management. It has become clear that integrating multiple tests in clinical practice not only increases patients’ chances of receiving appropriate and effective therapies but also underscores the paramount importance of developing novel and more precise biomarkers in the era of precision oncology.

## Figures and Tables

**Table 1:** The table lists seven Food and Drug Administration (FDA)-approved immune checkpoint inhibitors for first-line treatment of advanced NSCLC.

Drug Name	Target	Use in NSCLC
Pembrolizumab	PD-1	1st-line monotherapy (PD-L1 ≥ 1%), 1st line with chemotherapy, 2nd line after chemotherapy
Nivolumab	PD-1	2nd line after chemotherapy, 1st line in combination with ipilimumab ± chemotherapy
Atezolizumab	PD-L1	1st-line monotherapy (PD-L1 ≥ 50%) or in combination with bevacizumab + chemotherapy
Durvalumab	PD-L1	Consolidation for stage III unresectable NSCLC after chemoradiotherapy; 1st line in combination with tremelimumab + chemotherapy
Ipilimumab	CTLA-4	Only in combination with nivolumab
Cemiplimab	PD-1	1st-line monotherapy (PD-L1 ≥ 50%), 1st line with chemotherapy
Tremelimumab	CTLA-4	Only in combination with durvalumab + chemotherapy

**Table 2:** The table presents 13 major clinical trials evaluating immune checkpoint inhibitors (specifically PD-1 and PD-L1 inhibitors) in non-small cell lung cancer (NSCLC).

Trial Name	Clinical Trials.gov ID (Study Completion)	ICIs Evaluated	Comparator	Patient Population Enrollment (N)	Key Findings
KEYNOTE-407	NCT02775435 (September 2023)	Pembrolizumab (PD-1 inhibitor) + chemo	Platinum-based chemo	Squamous NSCLC (1st line) N = 559	Pembrolizumab + chemo significantly improved overall survival
OAK	NCT02008227 (January 2019)	Atezolizumab (PD-L1 inhibitor)	Docetaxel	Previously treated NSCLC N = 1225	Atezolizumab offered a survival benefit vs. docetaxel
IMpower150	NCT02366143 (February 2020)	Atezolizumab + chemo + bevacizumab	Chemo ± bevacizumab	1st-line metastatic non-squamous NSCLC N = 1202	Improved progression-free and overall survival with ICI combo.
CheckMate-9LA	NCT03215706 (October 2024)	Nivolumab (PD-1 inhibitor) + ipilimumab (CTLA-4 inhibitor) + chemo	Platinum-based chemo	1st line advanced NSCLC N = 719	Nivo + ipi + chemo significantly improved overall survival
CheckMate-227	NCT02477826 (October 2024)	Nivolumab + ipilimumab	Platinum-based chemo	Advanced NSCLC (1st line, various PD-L1 levels) N = 2748	Dual ICI therapy improved survival
POSEIDON	NCT03164616 (ongoing–November 2027)	Durvalumab (PD-L1 inhibitor) + tremelimumab (CTLA-4 inhibitor) + chemotherapy	Platinum-based chemo	NSCLC (1st line) N = 1186	Durva + treme + chemo significantly improved progression-free survival
